# Rickettsia-host interaction: strategies of intracytosolic host colonization

**DOI:** 10.1093/femspd/ftab015

**Published:** 2021-03-11

**Authors:** Oliver H Voss, M Sayeedur Rahman

**Affiliations:** Department of Microbiology and Immunology, University of Maryland School of Medicine, HSF2, room 416, 20 Penn St, Baltimore, MD 21201, USA; Department of Microbiology and Immunology, University of Maryland School of Medicine, HSF2, room 416, 20 Penn St, Baltimore, MD 21201, USA

**Keywords:** *Rickettsia-*host interaction, spotted fever group, transition group, typhus group, bacterial effector molecules, bacterial adherence and engulfment, phagosomal escape, phosphoinositide metabolism, intracellular trafficking, host defenses

## Abstract

Bacterial infection is a highly complex biological process involving a dynamic interaction between the invading microorganism and the host. Specifically, intracellular pathogens seize control over the host cellular processes including membrane dynamics, actin cytoskeleton, phosphoinositide metabolism, intracellular trafficking and immune defense mechanisms to promote their host colonization. To accomplish such challenging tasks, virulent bacteria deploy unique species-specific secreted effectors to evade and/or subvert cellular defense surveillance mechanisms to establish a replication niche. However, despite superficially similar infection strategies, diverse *Rickettsia* species utilize different effector repertoires to promote host colonization. This review will discuss our current understandings on how different *Rickettsia* species deploy their effector arsenal to manipulate host cellular processes to promote their intracytosolic life within the mammalian host.

## Teaser

Rickettsiae deploy their effector arsenal to manipulate membrane dynamics, actin cytoskeleton, phosphoinositide metabolism, intracellular trafficking and immune defense mechanisms; to gain access, and promote their intracytosolic lifespan to ultimately expedite transmission.

## INTRODUCTION

Rickettsiae host cell invasion is a dynamic process that involves a complex interplay between the invading pathogens and the host. During invasion, intracellular bacteria generate regulatory control over host cells by modulating membrane dynamics, actin cytoskeleton, phosphoinositide (PI) metabolism, intracellular trafficking and immune defense mechanisms; to gain access, and promote their survival and proliferation to ultimately expedite transmission (Ray *et al*. 2009; Pizarro-Cerdá, Kühbacher and Cossart 2015; Personnic *et al*. 2016; Lamason and Welch 2017). After internalization, intracellular pathogens encounter innate defense surveillance initiated upon pathogen sensing or other danger signals in the host cytosol. Pathogenic bacteria have evolved multiple strategies with numerous effectors, to evade and/or subvert innate defense surveillance to successfully colonize the host (Huang and Brumell 2014; Mitchell and Isberg 2017). Akin to other intracytosolic bacteria, including *Listeria*, *Shigella, Burkholderia* and *Francisella* (Ray *et al*. 2009; Personnic *et al*. 2016), *Rickettsia* internalization by phagocytosis and subsequent escape of pathogens into host cytosol is required for the survival and ultimately colonization of the host cell (Hackstadt 1996; Hackstadt 1998; Gillespie *et al*. 2015; Gillespie *et al*. 2016; Sahni *et al*. 2018). Recent work in our laboratory and others have identified several rickettsial effectors that function during the early stage of host cell invasion. While several surface proteins characterized for adhesion and/or entry of host cell are conserved, others are only sporadically encoded across rickettsial lineages, suggesting that, despite superficially similar infection strategies, diverse *Rickettsia* species (spp) employ distinct biochemical mechanisms to enable host colonization (Sears *et al*. 2012; Rahman *et al*. 2013; Gillespie *et al*. 2015; Rennoll-Bankert *et al*. 2015; Gillespie *et al*. 2016; Lamason *et al*. 2016; Rennoll-Bankert *et al*. 2016; Sahni *et al*. 2018; Lehman *et al*. 2018; Engström *et al*. 2019; Voss *et al*. 2020; Aistleitner *et al*. 2020). In this review, we will discuss recent advances on our understanding of how diverse *Rickettsia* spp utilizes their unique effector arsenal to alter host dynamic and spatiotemporal biochemical processes to establish an intracytosolic replication niche.

## RICKETTSIA SPECIES

Members of the genus *Rickettsia*, belonging to the class of *Alphaproteobacteria*; in the family of *Rickettsiaceae* and order of *Rickettsiales*; are Gram-negative obligate intracellular bacteria, which can invade a wide range of eukaryotes, including blood- or sap-feeding arthropods. Based on molecular phylogeny estimation, *Rickettsia* spp are classified into four groups: spotted fever group (SFG), typhus group (TG), transitional group (TRG) and ancestral group (AG) (Gillespie *et al*. 2007; Gillespie *et al*. 2009; Gillespie *et al*. 2010). Among these four *Rickettsia* lineages, we have very little information about members of AG, while the remaining three lineages: SFG, TRG, and TG, that harbor many deadly human pathogens, are well studied (Gillespie *et al*. 2007; Walker and Ismail 2008; Weinert *et al*. 2009; Gillespie *et al*. 2010; Murray *et al*. 2016). *Rickettsia* from the SFG, like *R. rickettsii* and *R. conorii*, are maintained naturally within various species of ticks, while TRG members, such as *R. akari*, *R. australis*, and *R. felis*, are maintained and transmitted to humans respectively via mites, ticks and fleas (Fig. 1). In addition, species from the TG, such as *R. prowazekii* and *R. typhi*, can be found throughout the world in fleas and lice (Fig. 1). Intriguingly, regardless of the phyletic relationship only a selected few of *Rickettsia* spp are considered highly pathogenic by causing disease in humans, while others show little to no pathogenicity. In particular, rickettsial infections causing fatal disease in humans, include Rocky Mountain Spotted Fever [*R. rickettsii, Sheila Smith, (SS)*], Boutonneuse fever (*R. conorii*), rickettsial pox (*R. akari*), epidemic typhus (*R. prowazekii*), or murine typhus (*R. typhi*) (Fig. 1).

**Figure:**
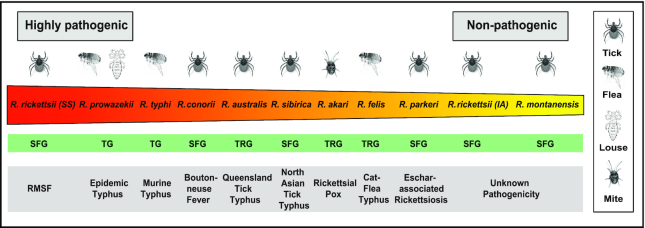
**Figure 1**. *Rickettsia* species-specific pathogenicity and disease development in humans. Natural transmission of rickettsiae to humans is accomplished by various arthropod vectors (ticks, fleas, lice, or mites) resulting in rickettsiosis with various degrees ranging from highly severe [*e.g*. Rocky Mountain Spotted Fever (RMSF)], to moderate (like cat-flea typhus), or ultimately asymptomatic (Hackstadt 1996; Hackstadt 1998; Azad and Beard 1998; Sahni *et al*. 2013; Clark *et al*. 2015; Curto *et al*. 2016). SFG: Spotted Fever Group; TRG: Transition Group; TG: Typhus Group; *R. rickettsii* [*Sheila Smith, (SS)*], *R. rickettsii* [*Iowa, (IA)*].

**Figure 2.**
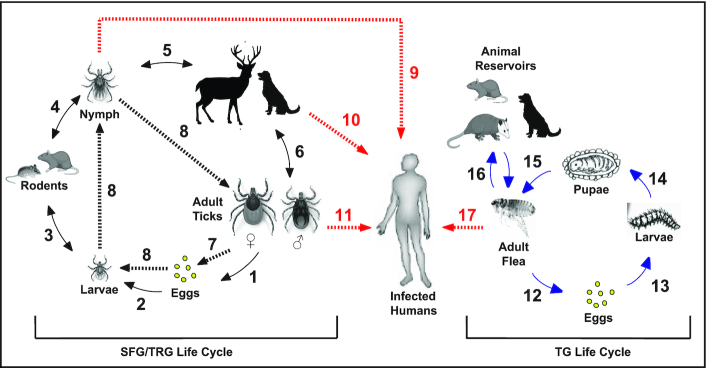
Life cycle of ticks and fleas and their natural transmission of rickettsiae. Black arrow lines emphasis key events of ticks natural cycle: (1) oviposition by engorged female ticks; (2) eggs hatched into larvae; (3) larvae feeding on rodents; (4) larvae molting to nymphs; (5) feeding of nymphs on larger animals; and (6) molting of nymphs into adult ticks that can feed on larger animals or infect humans (diagram was modified from (Eremeeva and Dasch 2015)). Broken black lines highlights transovarial (7) and transstadial transmission (8) of rickettsiae, while broken red lines show transmission of rickettsiae to humans through a direct bite of nymphs (9, 10) or adult ticks (11). Blue arrow lines emphasis key events of fleas natural cycle: (12) female fleas shed eggs into the environment (13) eggs hatched into larvae; (14) larvae form pupae; (15) pupae hatch to adult fleas; while transmission of rickettsiae to animals (16) or humans (broken red line, 17) occurs through inoculation of bacteria-laden flea feces onto flea bite wounds or mucous membranes (Azad *et al*. 1997; Anstead 2020).

Rickettsiae are zoonotic pathogens, with a worldwide distribution, that are transmitted to humans by the bite of arthropods (*e.g*. ticks) or via the feces of infected arthropods, like lice and fleas (Fig. 2) (Hackstadt 1996; Hackstadt 1998; Walker and Ismail 2008; Gillespie *et al*. 2009; Gillespie *et al*. 2015). Apart from the historical record, the global impact of arthropod-borne rickettsial infections is illustrated by the resurgence of long-known pathogens, as well as the emergence of newly recognized spp (Sanchez-Vicente *et al*. 2019). Infections of humans with *R. rickettsii* continues to cause severe consequences in South and Central America (Bermúdez and Troyo 2018), and the resurgence of *R. conorii* in Europe, the Middle East, and Africa further highlights the current threats of rickettsial diseases (Levin *et al*. 2009). In the USA, tick- and flea-borne rickettsial diseases are also on the rise, as exemplified by recent outbreaks of *R. rickettsii* in Arizona (Drexler *et al*. 2014) and of *R. typhi* in California (Billeter and Metzger 2017) and Texas (Blanton *et al*. 2016). There are currently no vaccines to prevent rickettsiosis. Additionally, a poor understanding of rickettsial intracellular lifestyle present an immense challenge to research and hinders progress towards development of effective intervention against these increasingly recognized rickettsioses (Sahni *et al*. 2018).

**Figure 3.**
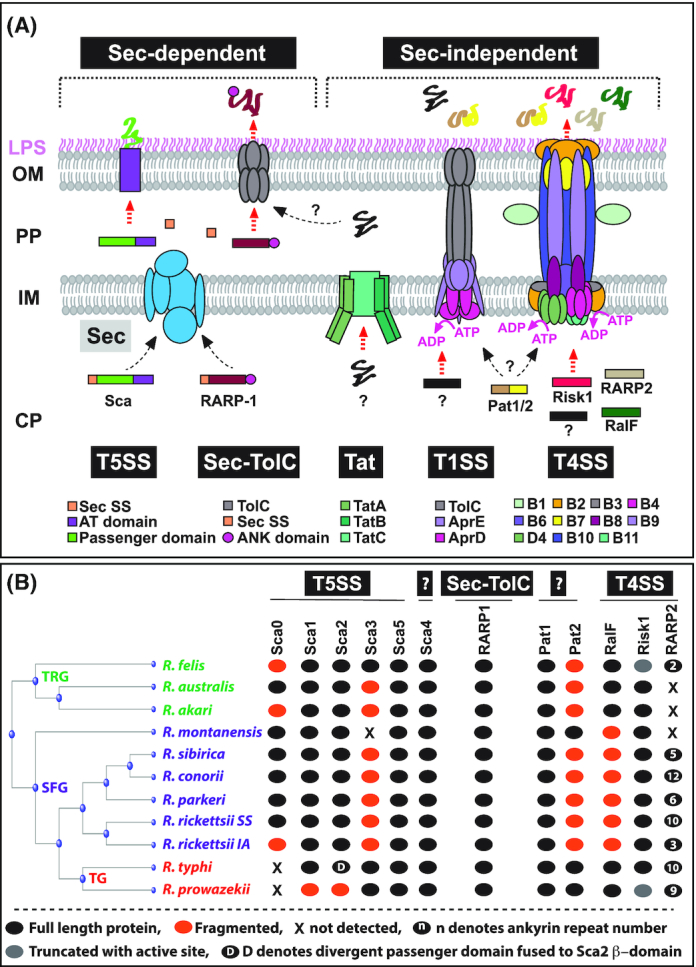
Effector section systems and distribution across divergent *Rickettsia* species. **(A)***Rickettsia* spp utilize two distinct secretory pathways (adopted and updated from our previous reporting (Gillespie *et al*. 2015)). The T5SS and Sec-TolC pathway are two Sec-dependent pathways. The T5SS system is considered to be involved in the secretion of the surface cell antigen (Sca) family. Currently, *Rickettsia* ankyrin repeat protein 1 (RARP-1) is the only effector secreted by the Sec-TolC system and involves its N-Terminal secretion signal (Sec SS), C-Terminal ankyrin (ANK) domain, and the TolC protein. The highly conserved sec-independent secretory pathways included the twin-arginine translocation (Tat), T1SS and T4SS systems. The Tat system is composed three components (TatA, TatB and TatC) and involved in the translocation of folded substrates across the inner membrane (IM). The TolC protein combined with additional IM proteins (AprE and AprD) form the functional T1SS system. The *Rickettsiales vir* homolog (*rvh*) T4SS, is highly similar to the *vir* structure of *Agrobacterium tumefaciens*, however with some difference including the duplication of several scaffold molecules and the lack of a pilus. CP: cytoplasm; PP: periplasm; OM: outer membrane; LPS: lipopolysaccharide; ?: unknown effectors or mechanism remains to be determined. **(B)** Phylogeny and effector molecules distribution across transitional group (TRG), spotted fever group (SFG) and typhus group (TG) *Rickettsia* spp (Rahman *et al*. 2010; Sears *et al*. 2012; Kaur *et al*. 2012; Rahman *et al*. 2013; Gillespie *et al*. 2015; Rennoll-Bankert *et al*. 2015; Gillespie *et al*. 2016; Lehman *et al*. 2018; Voss *et al*. 2020). 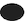 Full length protein; 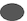 Truncated with active site; 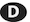 D denotes divergent passenger domain fused to Sca2 β-domain; 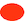 Fragmented; 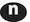 n denotes ankyrin repeat number within full length protein; **X** not detected.

**Figure 4.**
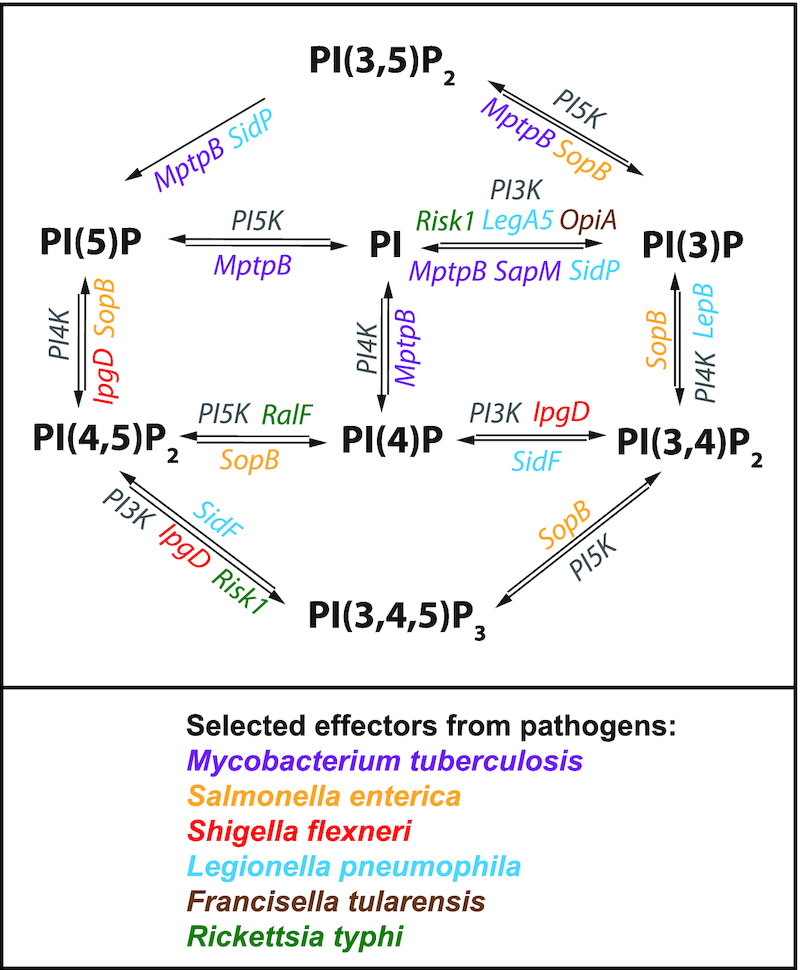
Repurposing of host phosphoinositides (PI) metabolism by intracellular bacterial effectors. The repurposing of host cell phosphoinositides (PI) is highly effective process of various intracellular pathogens to hijack intracellular trafficking and subvert host defense mechanisms to establish an intracellular niche (Pizarro-Cerdá *et al*. 2015; Walpole *et al*. 2018; Allen and Martinez 2020). Host enzymes for PI metabolism are shown in grey. Bacterial effectors that modulate host PI metabolism directly or indirectly are depicted in the corresponding colors (Niebuhr *et al*. 2002; Hernandez *et al*. 2004; Vergne *et al*. 2005; Pendaries *et al*. 2006; Beresford *et al*. 2007; Hsu *et al*. 2012; Toulabi *et al*. 2013; Rennoll-Bankert *et al*. 2015; Dong *et al*. 2016; Ledvina *et al*. 2018; Voss *et al*. 2020).

**Figure 5.**
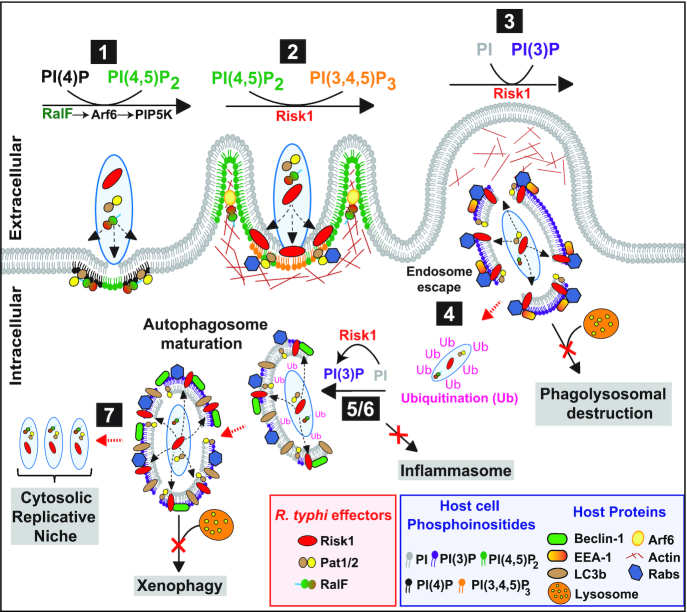
Model for host cell colonization mediated by effectors of the virulent *R. typhi* spp. Stage 1, RalF activates Arf6, which in turn recruits PIP5K to generate PI(4,5)P_2_ and to promote actin remodeling for pseudopodia formation (Rennoll-Bankert *et al*. 2015; Rennoll-Bankert *et al*. 2016); **Stage 2**, Risk1 promotes phagocytic uptake into host cells through the conversion of PI(4,5)P_2_ to PI(3,4,5)P_3_; **Stage 3**, Risk1 facilitates the generation of vacuolar PI(3)P to delay/subvert phagosomal maturation and allows rickettsial phospholipase effectors Pat1 and Pat2 (Pat1/2) to mediate bacterial escape into host cytosol; **Stage 4**, after phagosomal escape *Rickettsia/Rickettsia*-associated membrane remnants becomes ubiquitinated, resulting in the initiation of autophagy; **Stage 5**, Risk1 binds with Beclin-1, and facilitates generation of vacuolar PI(3)P, that (**Stage 6**) leads to delay/subvert autophagosomal maturation and likely the inhibition of inflammasome activation; **Stage 7**, the delay in autophagosomal maturation, allows Pat1 and Pat2 to mediate bacterial escape into host cytosol to establish replication niche (Rahman *et al*. 2013; Voss *et al*. 2020).

Rickettsiae infect a wide range of host cells where the metabolite-enriched host cytosol sustains their survival and growth in the face of reduced genomes that lack genes for many metabolic pathways (Driscoll *et al*. 2017). Rickettsial infection into the host begins with the inoculation of *Rickettsia* spp via infected arthropod vectors, like ticks, mites, lice or fleas, at the host's dermis (Fig. 2), where they encounter tissue-resident CD68^+^-macrophages (MΦ) and dendritic cells (Sahni *et al*. 2018). In fact, MΦ play a critical role in either terminating an infection at an early stage or succumbing to pathogen colonization and thus facilitating bacterial replication and host dissemination to distant organs, including lung, liver, heart and brain (Sahni *et al*. 2018). Given that intracytosolic survival depends on the escape from phagosomes and subversion of host cytosolic defense responses, in particular autophagy and inflammasomes, *Rickettsia* spp have develop sophisticated strategies to facilitate host invasion and to circumvent host immune defenses (discussed later in this review) (Ray *et al*. 2009; Personnic *et al*. 2016). Here, we will discuss the current advances of how virulent *Rickettsia* spp utilize their sec-dependent and -independent secretion pathways to translocate immunodominant outer membrane proteins [e.g. surface cell antigens (Scas)], actin modulating factor, RalF, the *Rickettsia* ankyrin repeat proteins (RARP-1/-2), *Rickettsia* intracellular secreted kinase-1 (Risk1), and other effectors [i.e. phospholipases (Pat1/Pat2) (Fig. 3) (Ammerman, Rahman and Azad 2008; Rahman *et al*. 2010; Sears *et al*. 2012; Kaur *et al*. 2012; Rahman *et al*. 2013; Gillespie *et al*. 2015; Rennoll-Bankert *et al*. 2015; Gillespie *et al*. 2016; Lehman *et al*. 2018; Voss *et al*. 2020), to target host PI metabolism and evade host defense surveillance to establish habitable intracytosolic replication niche (Figs 4 and 5).

## HOST INVASION: SURFACE CELL ANTIGEN PROTEINS

Despite their reductive genomes, *Rickettsia* spp are highly complex organisms that choreograph the expression of multiple surface proteins for successful entry and to establish a replication niche within a nutrient rich host cytosol. Host invasion of *Rickettsia* spp requires ligand engagement of specific receptors, as well as hijacking of specific host signaling cascades, which ultimately results in the rearrangements of actin cytoskeleton and membrane dynamics. Key molecules contributing to *Rickettsia* invasion are surface cell antigens (Scas), a class of immunodominant outer membrane (OM) proteins, for which nearly each spp of *Rickettsia* encodes a different Sca arsenal (Chan, Riley and Martinez 2010; Gillespie *et al*. 2015). Initial computation analysis of rickettsial genomes designated 17 distinct Sca family member (Blanc, Renesto and Raoult 2005) of which only 6 members are encoded in most rickettsial genomes: Sca0 (rOmpA), Sca1, Sca2, Sca3, Sca4 and Sca5 (rOmpB) (Sears *et al*. 2012; Gillespie *et al*. 2015). The secretion of Sca proteins is mediated by a sec-dependent type V secretion system (T5SS) to the OM of rickettsiae, except Sca4, a molecule which lacks the β-domain; and was extensively discussed in our earlier review (Gillespie *et al*. 2015) and is summarized in Fig. 3. Conserved in all rickettsiae spp, the ubiquitous **Sca5** (rOmpB) was shown to mediate bacterial invasion through its association with the host cell-specific receptor Ku70 (subunit of a nuclear DNA-dependent PK) (Martinez *et al*. 2005; Chan *et al*. 2009). In turn, association of Ku70 with rOmpB results in the recruitment of ubiquitin ligase c-Cbl (causing ubiquitination of Ku70), clathrin and caveolin-2 to facilitate the engulfment of the bacteria (Martinez *et al*. 2005). The less conserved **Sca0** (rOmpA), absent in most non-SFG spp (Fig. 3B), was also shown to be important for SFG rickettsiae internalization via its interaction with α2β1 integrin (Hillman, Baktash and Martinez 2013). However, further studies revealed that rOmpA was dispensable for the bacterial virulence *in vivo* (Noriea, Clark and, Hackstadt 2015). A very recent finding highlighted another host cell receptor FGFR1 (fibroblast growth factor receptor-1) as a potential target for rickettsial rOmpA to promote the internalization of SFG rickettsiae into host endothelium via a caveolin 1-dependent endocytosis (Sahni *et al*. 2017).

Genome analysis revealed the presence of full-length **Sca1** in all *Rickettsia* spp, except being divided in *R. prowazekii* (Fig. 3B) and *R. canadensis* (Ngwamidiba *et al*. 2006). Autotransporter Sca1 is expressed in *R. conorii* and *R. typhi* and was shown to play a role in the adherence of the bacteria to the target cell, however, its function during rickettsial invasion and the identity of its mammalian receptor remains elusive (Ngwamidiba *et al*. 2006; Riley *et al*. 2010; Sears *et al*. 2012). The surface cell antigen **Sca2** is ∼150 kDa protein and conserved among most SFG and TRG *Rickettsia* spp, while fragmented in *R. prowazekii* (Ngwamidiba *et al*. 2006; Dreher-Lesnick *et al*. 2008; Cardwell and Martinez 2009) (Fig. 3B). Sears *et al*. 2012 Sca2 was shown to be involved in the adherence to and engulfment of host cells (Cardwell and Martinez 2009), although its mammalian target receptor remains unknown. Autotransporter **Sca3** is exclusively encoded within the genomes of TG rickettsiae and flea-associated *R. felis* of TRG rickettsiae (Fig. 3B) and is considered as the largest rickettsial surface protein among the Sca family. However, Sca3 functional importance in the adherence to and engulfment of target cells remains to be uncovered (Sears *et al*. 2012). Genome sequence analysis showed the presence of **Sca4** in all rickettsial groups, including *R. prowazekii*, *R. typhi* (Fig. 3B) and *R. bellii*, while absent in *R. canadensis* (Blanc, Renesto and Raoult 2005; Sears *et al*. 2012). Intriguingly, Sca4, although lacking its autotransporter domain, is transported by a currently unknown mechanism to the rickettsial surface resulting in the activation of vinculin at the focal adhesion sites supporting a role for Sca4 in facilitating the invasion of target cells ( Blanc, Renesto and Raoult 2005; Park *et al*. 2011; Gillespie *et al*. 2015).

## HOST ACTIN CYTOSKELETON REARRANGEMENT AND CELL-TO-CELL SPREAD

As bacterial dissemination into neighboring cells is vital for a successful host colonization, rickettsial invasion involves host actin polymerization to form the typical F-actin ‘comet tail’. Notably, polar actin tail formation occurs exclusively in most SFG rickettsiae with the exception of *R. peacockii*, whereas minimal to no tail formation was observed among TG members (*R. typhi* and *R. prowazekii*) (Teysseire, Chiche-Portiche and Raoult 1992). As a result, infection of SGF rickettsiae initiates the activation of a signaling cascade involving Cdc42 (a GTPase), a phosphoinositide 3-kinases (PI3Ks), c-Src and likely additional protein tyrosine kinases, ultimately leading to the activation of Arp2/3 complex (Martinez and Cossart 2004). In fact, *in vitro* cell culture studies revealed that various *Rickettsia* spp from both SFG and TRG (*e.g. R. conorii*, *R. rickettsii*, *R. montanensis*, *R. australis, R. parkeri* and *R. felis*) utilize the effector **RickA** (absent in TG rickettsiae), a Wiskott–Aldrich syndrome protein (WASP), to promote actin-based motility and recruitment of the Arp2/3 complex (Heinzen *et al*. 1993; Gouin *et al*. 2004; Ogata *et al*. 2005; Balraj *et al*. 2008). Intriguingly, more recent findings support a model of biphasic rickettsial motility in which during early phase of infection (∼ 30 min) motility is dependent on RickA and the Arp2/3 complex, while upon persistent infection (∼24-48 hrs), motility is independent of Arp2/3 complex and RickA; and involves rickettsial Sca2 protein (Reed *et al*. 2014). Specifically, Sca2 nucleates the assembly of linear actin filaments, via its three WASP homology 2 (WH2) domains, resulting in the filament elongation by profilin and the inhibition capping protein activities (Haglund *et al*. 2010; Kleba *et al*. 2010; Lamason and Welch 2017).

Although, members of TG rickettsiae do not encode a functional orthologue of Sca2 (Fig. 3B), various spp of SFG and TRG rickettsiae; including *R. rickettsii*, *R. australis*, *R. conorii*, *R. africae*, and *R. akari*; encode Sca2, it is speculated that majority of SFG and TRG rickettsiae use the eukaryotic formin-like properties of Sca2 as a key mechanism for dissemination to neighboring host cells (Kleba *et al*. 2010; Gillespie *et al*. 2015). Recent report showed *R. parkeri* secretes an effector Sca4 that binds to the cell adhesion protein vinculin, inflicting the disruption of donor cell interaction between vinculin and α-catenin of recipient cell, which in turn promotes the cell to cell spread by enhancing protrusion engulfment efficiency (Lamason *et al*. 2016).

Another actin-binding molecule, **RalF**, expressed only in prokaryotes of some *Rickettsia* (Fig. 3B) and *Legionella* spp, is unique as it contains a N-terminal Sec7 domain (S7D) and C-terminal Sec7 capping domain (SCD) (Cox *et al*. 2004; Alix *et al*. 2012; Rennoll-Bankert *et al*. 2015). This domain is found among eukaryotic guanine nucleotide exchange factors (GEF) that activate ADP-ribosylation factors (Arfs), proteins involved in vesicle trafficking and actin remodeling (Casanova 2007). Both *Legionella* RalF (RalF_L_) and *Rickettsia* RalF (RalF_R_) function as ArfGEF that activates ArfGTPases by its catalytic S7D. However, RalF function differ significantly across these different pathogens and have divergent subcellular localization patterns mediated by the intrinsic determinants of SCD that confers distinct effector function in the host cells (Alix *et al*. 2012; Folly-Klan *et al*. 2013; Rennoll-Bankert *et al*. 2015; Rennoll-Bankert *et al*. 2016). *Legionella pneumophila* RalF (RalF_Lp_) binds and activates host Arf1 to localize the *Legionella*-containing vacuole (LCV) to the endoplasmic reticulum (ER) (Nagai *et al*. 2002; Nagai *et al*. 2005). The SCD of RalF_Lp_ likely contributes in the interception of host secretory vesicles, while that of *Rickettsia prowazekii* RalF (RalF_Rp_) targets the protein to the host PM to modulate actin dynamics (Alix *et al*. 2012; Folly-Klan *et al*. 2013). The role of SCD of RalF was investigated by chimera domain (S7D and SCD) swapping experiments between RalF_Lp_ and RalF_Rp_. The study revealed that the chimeric protein having S7D of RalF_Rp_ and SCD of RalF_Lp_ is as efficient in recruiting Arf1 to the LCV as wild type RalF_Lp_. However, the reverse chimeric protein having S7D of RalF_Lp_ and SCD of RalF_Rp_ was inefficient in mediating Arf1 recruitment to LCV (Alix *et al*. 2012). Furthermore, the SCD of both RalF_Lp_ and RalF_Rp_ auto-inhibit the catalytic S7D in solution, and a favorable membrane environments derepress ArfGEF activity in both proteins (Folly-Klan *et al*. 2013). The membrane sensor within the SCD of RalF_Lp_ and RalF_Rp_ revealed the differential enrichment in aromatic/charged residues determining distinct membrane localization that regulates ArfGEF activity (Folly-Klan *et al*. 2013). The investigation of ArfGEF activity on membranes through chimeric proteins containing S7D of RalF_Lp_ and SCD of RalF_Rp_ (LpRpRalF) or S7D of RalF_Rp_ and SCD of RalF_Lp_ (RpLpRalF) revealed that RpLpRalF activates Arf1. However, LpRpRalF was almost inactive, suggesting that a structural transition to the active form was blocked. The crystal structure analysis revealed the formation of serendipitous salt bridges between the residues of S7D of RalF_Lp_ and SCD of RalF_Rp_ in LpRpRalF chimera which was further highlighted as the cause of impaired LpRpRalF activity on the membranes, either by enforcing strong auto-inhibition or compromising the conformation of membrane bound LpRpRalF (Folly-Klan *et al*. 2015). Further follow up studies on rickettsial RalF (RalF_R_), allowed us to show that *R. typhi* RalF (RalF_Rt_) localized to the host PM (Fig. 5). Specifically, *R. typhi* RalF (RalF_Rt_) localization to the PM required recruitment of PI(4,5)P_2_, via the activation of Arf6, which in turn initiated actin cytoskeleton rearrangement that was critical for the invasion of *R. typhi* suggesting a role for this effector in modulating PI metabolism (discussed later in this review) (Rennoll-Bankert *et al*. 2015; Rennoll-Bankert *et al*. 2016).

## ANKYRIN-REPEAT-CONTAINING PROTEINS

One of the most conserved protein–protein interaction motifs in nature are ankyrin domains (Mosavi, Minor and Peng 2002), with ankyrin repeat-containing proteins (ARPs) playing key roles in the pathogenicity of intracellular bacteria (Pan *et al*. 2008; Al-Khodor *et al*. 2010). However, each ARP identified from intracellular pathogens like *Anaplasma*, *Ehrlichia*, *Legionella*, and *Orientia* seem to perform strain specific tasks, such as directly modulating gene transcription, manipulating vesicular trafficking, interrupting signaling pathways, or disrupting organelles (Pan *et al*. 2008; Zhu *et al*. 2009; Price *et al*. 2010; Rikihisa and Lin 2010; Yang *et al*. 2015; VieBrock *et al*. 2015). Despite that, all rickettsial genomes encode variable numbers of predicted ARPs their functional importance during pathogenesis remains ill-defined (Gillespie *et al*. 2015). The most conserved ARPs are **RARP-1** and **RARP-2** (Gillespie *et al*. 2015) with RARP-1 being a Sec-TolC secreted effector (Kaur *et al*. 2012), while RARP-2 is secreted by a type IV secretion system (T4SS) (Fig. 3) (Lehman *et al*. 2018). As rickettsial RARP-1 precise role remains to be determined, RARP-2 from *R. rickettsii* (SFG) but not from *R. typhi* (TG) was shown to target the ER and trans-Golgi network upon infection of host cells (fuab016-) suggesting a role for this effector in targeting cellular organization to facilitate host colonization likely in species-specific manner.

## MEMBRANOLYTIC EFFECTORS

Adherence and engulfment of rickettsiae into host cells is a relatively fast dynamic process. However, successful intracytosolic survival requires rickettsial evasion from host defense surveillance by lysosomal destruction. Soon after internalization *Rickettsia* spp avoid phago-lysosome fusion by escaping into host cytosol (Ray *et al*. 2009). Rickettsiae utilize their secretory membranolytic effector arsenal, including hemolysins and phospholipases, to disrupt the phagosomal membranes and gain access to host cytosol (Radulovic *et al*. 1999; Renesto *et al*. 2003; Whitworth *et al*. 2005; Rahman *et al*. 2010; Housley, Winkler and Audia 2011; Rahman *et al*. 2013). All *Rickettsia* genome analysis revealed the presence of membranolytic enzymes: TlyA, TlyC, Pld, Pat1 and Pat2 (Gillespie *et al*. 2015). Although the precise functional role of **TlyA** for rickettsiae pathogenicity remains unknown, a membranolytic activity of the rickettsial **TlyC** protein from the *R. typhi* spp was demonstrated (Radulovic *et al*. 1999). **Pld** (phospholipase D), encoded by the *pld* gene, is highly conserved in all sequenced rickettsial genomes (Gillespie *et al*. 2015). The phospholipase activity of Pld, that functions as a dimer, was demonstrated *in vitro* (Renesto *et al*. 2003; Whitworth *et al*. 2005) and in a *in vivo* model (guinea pig) of rickettsiosis (Driskell *et al*. 2009). Intriguingly, domain structure predictions of *R. typhi* Pld suggest that it likely localizes to the rickettsial OM via a secretory pathway similar to that of RARP-1 (Fig. 3) ( Ammerman, Rahman and Azad 2008; Gillespie *et al*. 2015).

The phospholipase A2 (PLA_2_)-like activities have long being proposed to facilitate rickettsial phagosomal escape as well as host cell entry and exit (Winkler and Miller 1982; Winkler and Daugherty 1989; Silverman *et al*. 1992; Ojcius *et al*. 1995; Walker, Feng and Popov 2001). The sequence analysis of available genomes of *Rickettsia* spp, identified a conserved patatin (Pat)-like PLA_2_ encoding molecule, **Pat1** within all *Rickettsia* genomes (Blanc, Renesto and Raoult 2005). A more recent report further characterized a second PLA_2_-encoding protein, **Pat2**, within the TG rickettsiae genomes, which was only sporadically present in SFG or TRG rickettsial genomes (Rahman *et al*. 2010; Rahman *et al*. 2013). The sequence analysis of Pat1 and Pat2 enzymes suggest that both proteins are likely secreted by either the T1SS or T4SS (Fig. 3) (Gillespie *et al*. 2015). Furthermore, we demonstrated that *R. typhi* Pat2 possesses a PLA_2_ activity and is secreted into the host cell cytoplasm during infection (Rahman *et al*. 2010). A later report further confirmed a similar PLA_2_ activity for the *R. prowazekii* Pat2 enzyme (Housley, Winkler and Audia 2011). In line with these findings, Pat1 also possesses a PLA_2_ activity and is secreted into host cell cytoplasm during infection (Rahman *et al*. 2013). Importantly, antibody neutralization of either Pat1 or Pat2 significantly reduced the survival of *R. typhi*, highlighting the roles for both proteins during bacterial infection (Rahman *et al*. 2013). Collectively, these data support that *R. typhi*, and likely *R. prowazekii*, use similar phospholipases during host infection, a mechanism that perhaps distinguishes TG rickettsiae from other *Rickettsia* spp.

## LIPID MODIFYING EFFECTORS

Many intracellular pathogens have developed diverse strategies to avoid recognition and subsequent destruction by host microbicidal defense mechanisms to establish a successful host colonization (Hybiske and Stephens 2008; Ray *et al*. 2009; Mitchell and Isberg 2017; Sahni *et al*. 2018). In fact, after internalization into host cells, some intracellular bacteria such as *Shigella, Francisella*, and *Rickettsia* spp, escape phagosomal maturation into cytosol to evade lysosomal destruction; while others like *Mycobacterium*, *Salmonella*, and *Legionella* modify the vacuolar compartment, to create an intracellular replication niche (Ray *et al*. 2009; Pizarro-Cerdá, Kühbacher and Cossart 2015; Personnic *et al*. 2016). To accomplish such delicate tasks, all intracellular pathogens employ numerous effectors to commander the host PI metabolism by selectively manipulating different PI interconversion pathways (Fig. 4) (Pizarro-Cerdá, Kühbacher and Cossart 2015; Mitchell and Isberg 2017; Huang and Brumell 2014; Walpole, Grinstein and Westman 2018). Specifically, bacterial effectors can modulate PI interconversion by either directly act as eukaryotic-like PI kinase and phosphatase or indirectly functioning as regulator of host PI kinases and phosphatases (Fig. 4). *Legionella pneumophila* secretes PI4-Kinase LepB and the 3-phosphatase SidF, two *dot*/*icm* T4SS effectors that contribute to the synthesis of PI(4)P on the *Legionella*-containing vacuole to avoids endolysosomal destruction and to create a replication permissive vacuolar compartment (Fig. 4) (Hsu *et al*. 2012; Dong *et al*. 2016). In addition, *L. pneumophila* secretes another *dot*/*icm* effector, LegA5, a class III PI3-Kinase, consistent with numerous PI-interacting *dot*/*icm* effectors functioning to establish an intracellular vacuolar niche for *Legionella* (Fig. 4) (Ledvina *et al*. 2018; Steiner, Weber and Hilbi 2018). The intracytosolic bacteria, *Francisella tularensis*, secretes the T6SS effector OpiA, a PI3-Kinase, to enhance the production of PI(3)P on the *Francisella-*containing phagosome, which consequently prevents endolysosomal fusion and promotes the escape of the bacteria into host cytosol (Fig. 4) (Ledvina *et al*. 2018). As obligate intracytosolic bacteria, *Rickettsia* invasion into target cells also involves the manipulation of PI-metabolism and the evasion of lysosomal destruction (Ray *et al*. 2009, Pizarro-Cerdá, Kühbacher and Cossart 2015; Walpole, Grinstein and Westman 2018). In this effort, we reported that secretion of *R. typhi* T4SS effector, RalF, was critical for host cell invasion (Fig. 4) (Rennoll-Bankert *et al*. 2015). Particularly, we showed that RalF was expressed early during *R. typhi* infection, colocalized with the PM and activated Arf6, which in turn recruited the host PIP5-Kinase to facilitate the conversion of PI(4)P to PI(4,5)P_2_ (Fig. 5, Stage 1). These findings likely support a mechanism by which *R. typhi* RalF indirectly modulates the PI metabolism to facilitate bacterial host invasion (Rennoll-Bankert *et al*. 2015; Rennoll-Bankert *et al*. 2016). In our recent report (Voss *et al*. 2020), we showed the presence of an additional *R. typhi* T4SS effector, Risk1, targeting host PI metabolism during invasion (Fig. 4). Our informatic, biochemical and enzymatic analysis further characterized Risk1 as a PI3K with a substrate specificity for both PI and PI(4,5)P_2_, making it the first bacterial PI3K with both class I and class III activities (Voss *et al*. 2020). In addition, our findings support that Risk1-dependent PI3K activity was involved in the synthesis of PI(3)P and PI(3,4,5)P_3_ lipids critical for the engulfment of *R. typhi* into host cells and the subsequent escape from endolysosomal destruction (Fig. 5, Stages 2 and 3) (Rennoll-Bankert *et al*. 2016; Voss *et al*. 2020). Our data further suggest that Risk1 likely modulates *R. typhi*-induced autophagy through its association with Beclin-1 and contributes to the escape from autolysosomal destruction via the conversion of PI to PI(3)P on the vacuole (Fig. 5, Stages 4 to 6) (Voss *et al*. 2020). As PI(3)P consumption on either endosomal or autophagosomal membranes is required for their fusion with lysosomes, it is tempting to propose a conceptual model of *R. typhi* intracytosolic infection by which Risk1-dependent generation of PI(3)P on both the phagosomal and autophagosomal membranes results in the delay of their maturation. As a result, the delay in maturation of these structures would allow additional effectors, such as Pat1 and Pat2 phospholipases, to perforate their membranes to facilitate the escape of the rickettsiae into the host cytosol (Fig. 5, Stage 7).

Aside *R. typhi*, strategies to induce autophagy and subsequent exploitation of autophagosomes to enhance host invasion are shared among some other rickettsial members (*e.g. R. australis* (Bechelli *et al*. 2018)) and their relatives, including *Anaplasma phagocytophilum* and *Ehrlichia chaffeensis* (Rikihisa 2017). Also, *R. typhi* ability to delay autophagic maturation via secretion of an effector protein, is another layer of sophistication that is shared among various intracellular pathogens, including *Mycobacterium marinum* (Romagnoli *et al*. 2012), *Chlamydia trachomatis* (Yasir *et al*. 2011; Al-Younes *et al*. 2011), *Yersinia pestis* (Pujol *et al*. 2009), and *Francisella tularensis* (Asare and Kwaik 2011). However, it is important to note that a previous postulated model of ‘nutritional virulence’, in which autophagosomal cargo is rerouted to the vacuoles harboring these bacteria, is harder to envision for Rickettsia spp, as all bacteria exclusively replicate in the intracytosolic space and not within modified phagosomes. Intriguingly, *R. parkeri*, a mildly-virulent member of SFG, was very recently shown to avoid autophagy induction and to evade autophagic recognition through its surface protein rOmpB (Engström *et al*. 2019). On the contrary, the virulent TRG member, *R. australis*, benefited from *Atg5*-dependent autophagy induction and suppression of inflammasome-dependent IL-1β production to colonize the host (Bechelli *et al*. 2018). In fact, recent reports further suggest that autophagy can act on intracellular microbes upstream of the inflammasome and thereby functions as a negative regulator by degrading inflammasome components (Mitchell and Isberg 2017; Sun *et al*. 2017). Thus, it is possible that virulent *Rickettsia* spp, like *R. australis* and *R. typhi*, employ different effector-mediated mechanisms to induce autophagy that allows subversion of inflammasome-dependent recognition and to facilitate their host colonization, as compared to mildly- or non-virulent spp (e.g*. R. parkeri* and *R. montanensis*), however the precise mechanism remains to be determined. In sum, these data for diverse rickettsial spp accentuate the divergent strategies utilized across Rickettsiales for intracellular parasitism and additional research is required to elucidate the mechanisms on how these parasites modulate intracellular trafficking and manipulate host defense pathways to promote host invasion and intracytosolic replication.

## CONCLUSION AND FUTURE DIRECTION

As highlighted in this review significant lack of conservation is seen not only at the level of protein secretion systems or pathways but also among members of effector proteins. In fact, many effectors are either absent, truncated, fragmented or predicted as pseudogenes in one or more *Rickettsia* spp. Thus, aside surface molecules, secretory effector proteins are highly variable across *Rickettsia* spp. In fact, findings from our laboratory and others suggest that virulent *Rickettsia* spp, like *R. australis* and *R. typhi*, utilizes different effector-mediated mechanisms to hijack intracellular trafficking to subvert host defense pathways, like autophagy and inflammasomes, to facilitate host infection and dissemination, as compared to mildly- or non-virulent spp (e.g*. R. parkeri* and *R. montanensis*).

Future research is required to identify the precise mechanisms: (i) on how virulent *Rickettsia* spp utilize their effector repertoire to manipulate autophagic responses and (ii) by which effectors of virulent *Rickettsia* spp subvert inflammasome activation to establish intracytosolic replication niche and to promote host dissemination.

In sum, these studies will provide new insights on how pathogenic rickettsiae manipulate and evade host defenses, which ultimately will lead to the identification of a link that could be exploited for an anti-virulence strategy to develop better therapeutics to eradicate fatal rickettsial diseases.

## References

[bib1] Aistleitner K , Clark T, Dooley C et al. Selective Fragmentation of the Trans-Golgi Apparatus by Rickettsia Rickettsii. PLoS Pathog. 2020;16:1–21. 10.1371/journal.ppat.1008582 PMC7259798 32421751

[bib3] Al-Khodor S , Price CT, Kalia A et al. Functional Diversity of Ankyrin Repeats in Microbial Proteins. Trends Microbiol. 2010;18:132–9. 19962898 10.1016/j.tim.2009.11.004 PMC2834824

[bib5] Al-Younes HM , Al-Zeer MA, Khalil H et al. Autophagy-Independent Function of MAP-LC3 during Intracellular Propagation of Chlamydia Trachomatis. Autophagy. 2011;7:814–28. 21464618 10.4161/auto.7.8.15597

[bib2] Alix E , Chesnel L, Bowzard BJ et al. The Capping Domain in RalF Regulates Effector Functions. PLoS Pathog. 2012;8:1–15. 10.1371/journal.ppat.1003012 PMC3499574 23166491

[bib4] Allen PE , Martinez JJ Modulation of Host Lipid Pathways by Pathogenic Intracellular Bacteria. Pathogens. 2020;9:1–22. 10.3390/pathogens9080614 PMC7460438 32731350

[bib6] Ammerman NC , Rahman MS, Azad AF. Characterization of Sec-Translocon-Dependent Extracytoplasmic Proteins of Rickettsia Typhi. J Bacteriol. 2008;190:6234–42. 18641131 10.1128/JB.00794-08 PMC2546805

[bib7] Anstead GM . History, Rats, Fleas, and Opossums: the Ascendency of Flea-Borne Typhus in the United States, 1910–1944. Tropical Med Infect Dis. 2020;5:1–31. 10.3390/tropicalmed5010037 PMC7157735 32121541

[bib8] Asare R , Kwaik YA. Exploitation of Host Cell Biology and Evasion of Immunity by Francisella Tularensis. Front Microbiol. 2011;1: 1–14. 10.3389/fmicb.2010.00145 PMC3109322 21687747

[bib9] Azad AF , Beard CB. Rickettsial Pathogens and Their Arthropod Vectors. Emerg Infect Dis. 1998;4:179–86. 9621188 10.3201/eid0402.980205 PMC2640117

[bib10] Azad AF , Radulovic S, Higgins JA et al. Flea-Borne Rickettsioses: ecologic Considerations. Emerg Infect Dis. 1997;3:319–27. 9284376 10.3201/eid0303.970308 PMC2627639

[bib11] Balraj P , Nappez C, Raoult D et al. Western-Blot Detection of RickA within Spotted Fever Group Rickettsiae Using a Specific Monoclonal Antibody. FEMS Microbiol Lett. 2008;286:257–62. 18657112 10.1111/j.1574-6968.2008.01283.x

[bib12] Bechelli J , Vergara L, Smalley C et al. Atg5 Supports Rickettsia Australis Infection in Macrophages In Vitro and In Vivo. Infect Immun. 2018;87:1–19. 10.1128/IAI.00651-18 PMC6300621 30297526

[bib13] Beresford N , Patel S, Armstrong J et al. MptpB, a Virulence Factor from Mycobacterium Tuberculosis, Exhibits Triple-Specificity Phosphatase Activity. Biochem J. 2007;406:13–18. 17584180 10.1042/BJ20070670 PMC1948985

[bib14] Bermúdez S , Troyo A. A Review of the Genus Rickettsia in Central America, *Res Rep Trop Med*. 2018;9:103–12. 30050361 10.2147/RRTM.S160951 PMC6047601

[bib15] Billeter SA , Metzger ME. Limited Evidence for Rickettsia Felis as a Cause of Zoonotic Flea-Borne Rickettsiosis in Southern California. J Med Entomol. 2017;54:4–7. 28082625 10.1093/jme/tjw179

[bib16] Blanc G , Ngwamidiba M, Ogata H et al. Molecular Evolution of Rickettsia Surface Antigens: evidence of Positive Selection. Mol Biol Evol. 2005;22:2073–83. 15972845 10.1093/molbev/msi199

[bib17] Blanc G , Renesto P, Raoult D. Phylogenic Analysis of Rickettsial Patatin-like Protein with Conserved Phospholipase A2 Active Sites. Ann N Y Acad Sci. 2005;1063:83–86. 16481495 10.1196/annals.1355.012

[bib18] Blanton LS , Idowu BM, Tatsch TN et al. Opossums and Cat Fleas: new Insights in the Ecology of Murine Typhus in Galveston, Texas. Am J Trop Med Hyg. 2016;95:457–61. 27273642 10.4269/ajtmh.16-0197 PMC4973200

[bib19] Cardwell MM , Martinez JJ. The Sca2 Autotransporter Protein from Rickettsia Conorii Is Sufficient to Mediate Adherence to and Invasion of Cultured Mammalian Cells. Infect Immun. 2009;77:5272–80. 19805531 10.1128/IAI.00201-09 PMC2786473

[bib20] Casanova JE . Regulation of Arf Activation: the Sec7 Family of Guanine Nucleotide Exchange Factors. Traffic. 2007;8:1476–85. 17850229 10.1111/j.1600-0854.2007.00634.x

[bib22] Chan YG-Y , Riley SP, Martinez JJ. Adherence to and Invasion of Host Cells by Spotted Fever Group Rickettsia Species. Front Microbiol. 2010;1:1–10. 21687751 10.3389/fmicb.2010.00139 PMC3109342

[bib21] Chan YGY , Cardwell MM, Hermanas TM et al. Rickettsial Outer-Membrane Protein B (ROmpB) Mediates Bacterial Invasion through Ku70 in an Actin, c-Cbl, Clathrin and Caveolin 2-Dependent Manner. Cell Microbiol. 2009;11:629–44. 19134120 10.1111/j.1462-5822.2008.01279.x PMC2773465

[bib23] Clark TR , Noriea NF, Bublitz DAC et al. Comparative Genome Sequencing of Rickettsia Rickettsii Strains That Differ in Virulence. Infect Immun. 2015;83:1568–76. 25644009 10.1128/IAI.03140-14 PMC4363411

[bib24] Cox R , Mason-Gamer RJ, Jackson CL et al. Phylogenetic Analysis of Sec7-Domain-Containing Arf Nucleotide Exchangers. Mol Biol Cell. 2004;15:1487–505. 14742722 10.1091/mbc.E03-06-0443 PMC379250

[bib25] Curto P , Simões I, Riley SP et al. Differences in Intracellular Fate of Two Spotted Fever Group Rickettsia in Macrophage-Like Cells. Front Cell Infect Microbiol. 2016;6:1–14. 27525249 10.3389/fcimb.2016.00080 PMC4965480

[bib26] Dong N , Niu M, Hu L et al. Modulation of Membrane Phosphoinositide Dynamics by the Phosphatidylinositide 4-Kinase Activity of the Legionella LepB Effector. Nat Microbiol. 2016;2:1–10. 10.1038/nmicrobiol.2016.236 27941800

[bib27] Dreher-Lesnick SM , Ceraul SM, Rahman MS et al. Genome-Wide Screen for Temperature-Regulated Genes of the Obligate Intracellular Bacterium, Rickettsia Typhi. BMC Microbiol. 2008;8:1–12. 18412961 10.1186/1471-2180-8-61 PMC2335108

[bib28] Drexler N , Miller M, Gerding J et al. Community-Based Control of the Brown Dog Tick in a Region with High Rates of Rocky Mountain Spotted Fever, 2012–2013. PLoS One. 2014;9:1–18. 10.1371/journal.pone.0112368 PMC4257530 25479289

[bib29] Driscoll TP , Verhoeve VI, Guillotte ML et al. Wholly *Rickettsia* ! Reconstructed Metabolic Profile of the Quintessential Bacterial Parasite of Eukaryotic Cells. *MBio*. 2017;8:1–27. 10.1128/mBio.00859-17 PMC5615194 28951473

[bib30] Driskell LO , Yu XJ, Zhang L et al. Directed Mutagenesis of the Rickettsia Prowazekii Pld Gene Encoding Phospholipase D. Infect Immun. 2009;77:3244–8. 19506016 10.1128/IAI.00395-09 PMC2715659

[bib31] Engström P , Burke TP, Mitchell G et al. Evasion of Autophagy Mediated by Rickettsia Surface Protein OmpB Is Critical for Virulence. Nat Microbiol. 2019;4:2538–51. 31611642 10.1038/s41564-019-0583-6 PMC6988571

[bib32] Eremeeva ME , Dasch GA. Challenges Posed by Tick-Borne Rickettsiae: eco-Epidemiology and Public Health Implications. Front Public Heal. 2015;3:1–17. 10.3389/fpubh.2015.00055 PMC4404743 25954738

[bib33] Folly-Klan M , Alix E, Stalder D et al. A Novel Membrane Sensor Controls the Localization and ArfGEF Activity of Bacterial RalF. PLoS Pathog. 2013;9:1–13. 10.1371/journal.ppat.1003747 PMC3828167 24244168

[bib34] Folly-Klan M , Sancerne B, Alix E et al. On the Use of Legionella/Rickettsia Chimeras to Investigate the Structure and Regulation of Rickettsia Effector RalF. J Struct Biol. 2015;189:98–104. 25498244 10.1016/j.jsb.2014.12.001

[bib35] Gillespie JJ , Ammerman NC, Beier-Sexton M et al. Louse- and Flea-Borne Rickettsioses: biological and Genomic Analyses. Vet Res. 2009;40:1–13. 19036234 10.1051/vetres:2008050 PMC2695025

[bib36] Gillespie JJ , Beier MS, Rahman MS et al. Plasmids and Rickettsial Evolution: insight from Rickettsia Felis. PLoS One. 2007;2:1–17. 10.1371/journal.pone.0000266 PMC1800911 17342200

[bib37] Gillespie JJ , Brayton KA, Williams KP et al. Phylogenomics Reveals a Diverse Rickettsiales Type IV Secretion System. 2010;78:1809–23. 10.1128/IAI.01384-09 PMC2863512 20176788

[bib38] Gillespie JJ , Kaur SJ, Sayeedur Rahman M et al. Secretome of Obligate Intracellular Rickettsia. FEMS Microbiol Rev. 2015;39:47–80. 25168200 10.1111/1574-6976.12084 PMC4344940

[bib39] Gillespie JJ , Phan IQH, Driscoll TP et al. The Rickettsia Type IV Secretion System: unrealized Complexity Mired by Gene Family Expansion. Pathog Dis. 2016;74. 10.1093/femspd/ftw058. PMC5505475 27307105

[bib40] Gouin E , Egile C, Dehoux P et al. The RickA Protein of Rickettsia Conorii Activates the Arp2/3 Complex. Nature. 2004;427:457–61. 14749835 10.1038/nature02318

[bib41] Hackstadt T . The Biology of Rickettsiae. Infect Agents Dis. 1996;5:127–43. 8805076

[bib42] Hackstadt T . The Diverse Habitats of Obligate Intracellular Parasites. Curr Opin Microbiol. 1998;1:82–87. 10066459 10.1016/s1369-5274(98)80146-x

[bib43] Haglund CM , Choe JE, Skau CT et al. Rickettsia Sca2 Is a Bacterial Formin-like Mediator of Actin-Based Motility. Nat Cell Biol. 2010;12:1057–63. 20972427 10.1038/ncb2109 PMC3136050

[bib44] Heinzen RA , Hayes SF, Peacock MG et al. Directional Actin Polymerization Associated with Spotted Fever Group Rickettsia Infection of Vero Cells. Infect Immun. 1993;61:1926–35. 8478082 10.1128/iai.61.5.1926-1935.1993 PMC280785

[bib45] Hernandez LD , Hueffer K, Wenk MR et al. Salmonella Modulates Vesicular Traffic by Altering Phosphoinositide Metabolism. Science. 2004;304:1805–7. 15205533 10.1126/science.1098188

[bib46] Hillman RD , Baktash YM, Martinez JJ. OmpA-Mediated Rickettsial Adherence to and Invasion of Human Endothelial Cells Is Dependent upon Interaction with Α2β1 Integrin. Cell Microbiol. 2013;15:727–41. 23145974 10.1111/cmi.12068 PMC3610814

[bib47] Housley NA , Winkler HH, Audia JP. The Rickettsia Prowazekii ExoU Homologue Possesses Phospholipase A 1 (PLA 1), PLA 2, and Lyso-PLA 2 Activities and Can Function in the Absence of Any Eukaryotic Cofactors in Vitro. J Bacteriol. 2011;193:4634–42. 21764940 10.1128/JB.00141-11 PMC3165714

[bib48] Hsu F , Zhu W, Brennan L et al. Structural Basis for Substrate Recognition by a Unique Legionella Phosphoinositide Phosphatase. Proc Natl Acad Sci USA. 2012;109:13567–72. 22872863 10.1073/pnas.1207903109 PMC3427105

[bib49] Huang J , Brumell JH. Bacteria-Autophagy Interplay: a Battle for Survival. Nat Rev Microbiol. 2014;12:101–14. 24384599 10.1038/nrmicro3160 PMC7097477

[bib50] Hybiske K , Stephens RS. Exit Strategies of Intracellular Pathogens. Nat Rev Microbiol. 2008;6:99–110. 18197167 10.1038/nrmicro1821

[bib51] Kaur SJ , Sayeedur Rahman M, Ammerman NC et al. TolC-Dependent Secretion of an Ankyrin Repeat-Containing Protein of Rickettsia Typhi. J Bacteriol. 2012;194:4920–32. 22773786 10.1128/JB.00793-12 PMC3430354

[bib52] Kleba B , Clark TR, Lutter EI et al. Disruption of the Rickettsia Rickettsii Sca2 Autotransporter Inhibits Actin-Based Motility. Infect Immun. 2010;78:2240–7. 20194597 10.1128/IAI.00100-10 PMC2863521

[bib53] Lamason RL , Bastounis E, Kafai NM et al. Rickettsia Sca4 Reduces Vinculin-Mediated Intercellular Tension to Promote Spread. Cell. 2016;167:670–683.e10. 27768890 10.1016/j.cell.2016.09.023 PMC5097866

[bib54] Lamason RL , Welch MD. Actin-Based Motility and Cell-to-Cell Spread of Bacterial Pathogens. Curr Opin Microbiol. 2017:35:48–57. 27997855 10.1016/j.mib.2016.11.007 PMC5474209

[bib55] Ledvina HE , Kelly KA, Eshraghi A et al. A Phosphatidylinositol 3-Kinase Effector Alters Phagosomal Maturation to Promote Intracellular Growth of Francisella. Cell Host Microbe. 2018;24:285–295.e8. 30057173 10.1016/j.chom.2018.07.003 PMC6394229

[bib56] Lehman SS , Noriea NF, Aistleitner K et al. The Rickettsial Ankyrin Repeat Protein 2 Is a Type IV Secreted Effector That Associates with the Endoplasmic Reticulum. MBio. 2018;9:1–15. 10.1128/mBio.00975-18 PMC6020290 29946049

[bib57] Levin ML , Killmaster L, Zemtsova G et al. Incongruent Effects of Two Isolates of Rickettsia Conorii on the Survival of Rhipicephalus Sanguineus Ticks. Exp Appl Acarol. 2009;49:347–59. 19421877 10.1007/s10493-009-9268-9

[bib58] Martinez JJ , Cossart P. Early Signaling Events Involved in the Entry of Rickettsia Conorii into Mammalian Cells. J Cell Sci. 2004;117:5097–106. 15383620 10.1242/jcs.01382

[bib59] Martinez JJ , Seveau S, Veiga E et al. Ku70, a Component of DNA-Dependent Protein Kinase, Is a Mammalian Receptor for Rickettsia Conorii. Cell. 2005;123:1013–23. 16360032 10.1016/j.cell.2005.08.046

[bib60] Mitchell G , Isberg RR. Innate Immunity to Intracellular Pathogens: balancing Microbial Elimination and Inflammation. Cell Host and Microbe. 2017;22:166–75. 28799902 10.1016/j.chom.2017.07.005 PMC5562164

[bib61] Mosavi LK , Minor DL, Peng Z-y. Consensus-Derived Structural Determinants of the Ankyrin Repeat Motif. Proc Natl Acad Sci USA. 2002;99:16029–34. 12461176 10.1073/pnas.252537899 PMC138559

[bib62] Murray GGR , Weinert LA, Rhule EL et al. The Phylogeny of Rickettsia Using Different Evolutionary Signatures: how Tree-Like Is Bacterial Evolution?. Syst Biol. 2016;65:265–79. 26559010 10.1093/sysbio/syv084 PMC4748751

[bib63] Nagai H , Cambronne ED, Kagan JC et al. A C-Terminal Translocation Signal Required for Dot/Icm-Dependent Delivery of the Legionella RalF Protein to Host Cells. Proc Natl Acad Sci USA. 2005;102:826–31. 15613486 10.1073/pnas.0406239101 PMC545534

[bib64] Nagai H , Kagan JC, Zhu X et al. A Bacterial Guanine Nucleotide Exchange Factor Activates ARF on Legionella Phagosomes. *Science*. 2002;295:679–82. 11809974 10.1126/science.1067025

[bib65] Ngwamidiba M , Blanc G, Raoult D et al. Sca1, a Previously Undescribed Paralog from Autotransporter Protein-Encoding Genes in Rickettsia Species. *BMC Microbiol*. 2006;6:1–11. 16504018 10.1186/1471-2180-6-12 PMC1388218

[bib66] Niebuhr K , Giuriato S, Pedron T et al. Conversion of PtdIns(4, 5)P2 into PtdIns(5)P by the S.Flexneri Effector IpgD Reorganizes Host Cell Morphology. EMBO J. 2002;21:5069–78. 12356723 10.1093/emboj/cdf522 PMC129044

[bib67] Noriea NF , Clark TR, Hackstadt T. Targeted Knockout of the Rickettsia Rickettsii OmpA Surface Antigen Does Not Diminish Virulence in a Mammalian Model System. MBio. 2015;6:1–9. 10.1128/mBio.00323-15 PMC4453529 25827414

[bib68] Ogata H , Renesto P, Audic S et al. The Genome Sequence of Rickettsia Felis Identifies the First Putative Conjugative Plasmid in an Obligate Intracellular Parasite. PLoS Biol. 2005;3:1391–402. 10.1371/journal.pbio.0030248 PMC1166351 15984913

[bib69] Ojcius DM , Thibon M, Mounier C et al. PH and Calcium Dependence of Hemolysis Due to Rickettsia Prowazekii: comparison with Phospholipase Activity. Infect Immun. 1995;63:3069–72. 7622232 10.1128/iai.63.8.3069-3072.1995 PMC173418

[bib70] Pan X , Lührmann A, Satoh A et al. Ankyrin Repeat Proteins Comprise a Diverse Family of Bacterial Type IV Effectors. Science. 2008;320:1651–4. 18566289 10.1126/science.1158160 PMC2514061

[bib71] Park HJ , Lee JH, Gouin E et al. The Rickettsia Surface Cell Antigen 4 Applies Mimicry to Bind to and Activate Vinculin. J Biol Chem. 2011;286:35096–103. 21841197 10.1074/jbc.M111.263855 PMC3186400

[bib72] Pendaries C , Tronchère H, Arbibe L et al. PtdIns(5)P Activates the Host Cell PI3-Kinase/Akt Pathway during Shigella Flexneri Infection. EMBO J. 2006;25:1024–34. 16482216 10.1038/sj.emboj.7601001 PMC1409730

[bib73] Personnic N , Bärlocher K, Finsel I et al. Subversion of Retrograde Trafficking by Translocated Pathogen Effectors. Trends Microbiol. 2016;24:450–62. 26924068 10.1016/j.tim.2016.02.003

[bib74] Pizarro-Cerdá J , Kühbacher A, Cossart P. Phosphoinositides and Host–Pathogen Interactions. Biochim Biophys Acta - Mol Cell Biol Lipids. 2015;1851:911–8. 10.1016/j.bbalip.2014.09.011 25241942

[bib75] Pizarro-Cerdá J , Kühbacher A, Cossart P Phosphoinositides and Host-Pathogen Interactions. 2015;1851:911–8. 10.1016/j.bbalip.2014.09.011 25241942

[bib76] Price CTD , Jones SC, Amundson KE et al. Host-Mediated Post-Translational Prenylation of Novel Dot/Icm-Translocated Effectors of Legionella Pneumophila. Front Microbiol. 2010;1:1–9. 21687755 10.3389/fmicb.2010.00131 PMC3109360

[bib77] Pujol C , Klein KA, Romanov GA et al. Yersinia Pestis Can Reside in Autophagosomes and Avoid Xenophagy in Murine Macrophages by Preventing Vacuole Acidification. Infect Immun. 2009;77:2251–61. 19289509 10.1128/IAI.00068-09 PMC2687347

[bib78] Radulovic S , Troyer JM, Beier MS et al. Identification and Molecular Analysis of the Gene Encoding Rickettsia Typhi Hemolysin. Infect Immun. 1999;67:6104–8. 10531273 10.1128/iai.67.11.6104-6108.1999 PMC96999

[bib79] Rahman MS , Ammerman NC, Sears KT et al. Functional Characterization of a Phospholipase A2 Homolog from Rickettsia Typhi. J Bacteriol. 2010;192:3294–303. 20435729 10.1128/JB.00155-10 PMC2897650

[bib80] Rahman MS , Gillespie JJ, Kaur SJ et al. Rickettsia Typhi Possesses Phospholipase A2 Enzymes That Are Involved in Infection of Host Cells. PLoS Pathog. 2013;9:1–17. 10.1371/journal.ppat.1003399 PMC3688537 23818842

[bib81] Ray K , Marteyn B, Sansonetti PJ et al. Life on the inside: the Intracellular Lifestyle of Cytosolic Bacteria. Nat Rev Microbiol. 2009;7:333–40. 19369949 10.1038/nrmicro2112

[bib82] Reed SCO , Lamason RL, Risca VI et al. Actin-Based Motility Occurs in Distinct Phases Mediated by Different Actin Nucleators. Curr Biol. 2014;24:98–103. 24361066 10.1016/j.cub.2013.11.025 PMC3951146

[bib83] Renesto P , Dehoux P, Gouin E et al. Identification and Characterization of a Phospholipase D-Superfamily Gene in Rickettsiae. J Infect Dis. 2003;188:1276–83. 14593584 10.1086/379080

[bib84] Rennoll-Bankert KE , Rahman MS, Gillespie JJ et al. Which Way In? The RalF Arf-GEF Orchestrates Rickettsia Host Cell Invasion. PLoS Pathog. 2015;11:1–28. 10.1371/journal.ppat.1005115 PMC4546372 26291822

[bib85] Rennoll-Bankert KE , Rahman MS, Guillotte ML et al. RalF-Mediated Activation of Arf6 Controls Rickettsia Typhi Invasion by Co-Opting Phosphoinositol Metabolism. Infect Immun. 2016;84:3496–506. 27698019 10.1128/IAI.00638-16 PMC5116726

[bib87] Rikihisa Y , Lin M. Anaplasma Phagocytophilum and Ehrlichia Chaffeensis Type IV Secretion and Ank Proteins. Curr Opin Microbiol. 2010;13:59–66. 20053580 10.1016/j.mib.2009.12.008 PMC3251840

[bib86] Rikihisa Y . Role and Function of the Type IV Secretion System in Anaplasma and Ehrlichia Species. Curr Top Microbiol Immunol. 2017;413:297–321. 29536364 10.1007/978-3-319-75241-9_12

[bib88] Riley SP , Goh KC, Hermanas TM et al. The Rickettsia Conorii Autotransporter Protein Sca1 Promotes Adherence to Nonphagocytic Mammalian Cells. Infect Immun. 2010;78:1895–904. 20176791 10.1128/IAI.01165-09 PMC2863548

[bib89] Romagnoli A , Etna MP, Giacomini E et al. ESX-1 Dependent Impairment of Autophagic Flux by Mycobacterium Tuberculosis in Human Dendritic Cells. Autophagy. 2012;8:1357–70. 22885411 10.4161/auto.20881 PMC3442882

[bib90] Sahni A , Fang R, Sahni SK et al. Pathogenesis of Rickettsial Diseases: pathogenic and Immune Mechanisms of an Endotheliotropic Infection. Annu Rev Pathol Mech Dis. 2018;14:127–52. 10.1146/annurev-pathmechdis-012418-012800 PMC6505701 30148688

[bib91] Sahni A , Patel J, Narra HP et al. Fibroblast Growth Factor Receptor-1 Mediates Internalization of Pathogenic Spotted Fever Rickettsiae into Host Endothelium. PLoS One. 2017;12:1–16. 10.1371/journal.pone.0183181 PMC5555671 28806774

[bib92] Sahni SK , Narra HP, Sahni A et al. Recent Molecular Insights into Rickettsial Pathogenesis and Immunity. Futur Microbiol. 2013;8:1265–88. 10.2217/fmb.13.102 PMC3923375 24059918

[bib93] Sanchez-Vicente S , Tagliafierro T, Coleman JL et al. Polymicrobial Nature of Tick-Borne Diseases. MBio. 2019;10:1–17. 10.1128/mBio.02055-19 PMC6737246 31506314

[bib94] Sears KT , Ceraul SM, Gillespie JJ et al. Surface Proteome Analysis and Characterization of Surface Cell Antigen (Sca) or Autotransporter Family of Rickettsia Typhi. PLoS Pathog. 2012;8:1–17. 10.1371/journal.ppat.1002856 PMC3415449 22912578

[bib95] Silverman DJ , Santucci LA, Meyers N et al. Penetration of Host Cells by Rickettsia Rickettsii Appears to Be Mediated by a Phospholipase of Rickettsial Origin. Infect Immun. 1992;60:2733–40. 1612741 10.1128/iai.60.7.2733-2740.1992 PMC257228

[bib96] Steiner B , Weber S, Hilbi H. Formation of the Legionella-Containing Vacuole: phosphoinositide Conversion, GTPase Modulation and ER Dynamics. Int J Med Microbiol. 2018;308:49–57. 28865995 10.1016/j.ijmm.2017.08.004

[bib97] Sun Q , Fan J, Billiar TR et al. Inflammasome and Autophagy Regulation: a Two-Way Street. Mol Med. 2017;23:188–95. 28741645 10.2119/molmed.2017.00077 PMC5588408

[bib98] Teysseire N , Chiche-Portiche C, Raoult D. Intracellular Movements of Rickettsia Conorii Adn R. Typhi Based on Actin Polymerization. Res Microbiol. 1992;143:821–9. 1299836 10.1016/0923-2508(92)90069-z

[bib99] Toulabi L , Wu X, Cheng Y et al. Identification and Structural Characterization of a Legionella Phosphoinositide Phosphatase. J Biol Chem. 2013;288:24518–27. 23843460 10.1074/jbc.M113.474239 PMC3750150

[bib100] Vergne I , Chua J, Lee HH et al. Mechanism of Phagolysosome Biogenesis Block by Viable Mycobacterium Tuberculosis. Proc Natl Acad Sci USA. 2005;102:4033–8. 15753315 10.1073/pnas.0409716102 PMC554822

[bib101] VieBrock L , Evans SM, Beyer AR et al. Orientia Tsutsugamushi Ankyrin Repeat-Containing Protein Family Members Are Type 1 Secretion System Substrates That Traffic to the Host Cell Endoplasmic Reticulum. *Front*. *Cell Infect Microbiol*. 2015;4:1–20. 10.3389/fcimb.2014.00186 PMC4315096 25692099

[bib102] Voss OH , Gillespie JJ, Lehman SS et al. Risk1, a Phosphatidylinositol 3-Kinase Effector, Promotes Rickettsia Typhi Intracellular Survival. *MBio*. 2020;11:1–22. 10.1128/mBio.00820-20 PMC7298712 32546622

[bib103] Walker DH , Feng HM, Popov VL. Rickettsial Phospholipase A2 as a Pathogenic Mechanism in a Model of Cell Injury by Typhus and Spotted Fever Group Rickettsiae. Am J Trop Med Hyg. 2001;65:936–42. 11792002 10.4269/ajtmh.2001.65.936

[bib104] Walker DH , Ismail N. Emerging and Re-Emerging Rickettsioses: endothelial Cell Infection and Early Disease Events. Nat Rev Microbiol. 2008;6:375–86. 18414502 10.1038/nrmicro1866

[bib105] Walpole GFW , Grinstein S, Westman J. The Role of Lipids in Host-Pathogen Interactions. IUBMB Life. 2018;70:384–92. 29573124 10.1002/iub.1737

[bib106] Weinert LA , Werren JH, Aebi A et al. Evolution and Diversity of Rickettsia Bacteria. BMC Biol. 2009;7:1–15. 19187530 10.1186/1741-7007-7-6 PMC2662801

[bib107] Whitworth T , Popov VL, Yu XJ et al. Expression of the Rickettsia Prowazekii Pld or TlyC Gene in Salmonella Enterica Serovar Typhimurium Mediates Phagosomal Escape. Infect Immun. 2005;73:6668–73. 16177343 10.1128/IAI.73.10.6668-6673.2005 PMC1230948

[bib108] Winkler HH , Daugherty RM. Phospholipase A Activity Associated with the Growth of Rickettsia Prowazekii in L929 Cells. Infect Immun. 1989;57:36–40. 2491840 10.1128/iai.57.1.36-40.1989 PMC313037

[bib109] Winkler HH , Miller ET. Phospholipase A and the Interaction of Rickettsia Prowazekii and Mouse Fibroblasts (L-929 Cells). Infect Immun. 1982;38:109–13. 6815087 10.1128/iai.38.1.109-113.1982 PMC347704

[bib110] Yang Q , Stevenson HL, Scott MJ et al. Type I Interferon Contributes to Noncanonical Inflammasome Activation, Mediates Immunopathology, and Impairs Protective Immunity during Fatal Infection with Lipopolysaccharide-Negative Ehrlichiae. Am J Pathol. 2015;185:446–61. 25481711 10.1016/j.ajpath.2014.10.005 PMC4305182

[bib111] Yasir M , Pachikara ND, Bao X et al. Regulation of Chlamydial Infection by Host Autophagy and Vacuolar ATPase-Bearing Organelles. Infect Immun. 2011;79:4019–28. 21807906 10.1128/IAI.05308-11 PMC3187247

[bib112] Zhu B , Nethery KA, Kuriakose JA et al. Nuclear Translocated Ehrlichia Chaffeensis Ankyrin Protein Interacts with a Specific Adenine-Rich Motif of Host Promoter and Intronic Alu Elements. Infect Immun. 2009;77:4243–55. 19651857 10.1128/IAI.00376-09 PMC2747939

